# Luminescence behaviour of nitrogen-doped graphene quantum dots *via* solvent variation for ferric ion sensing

**DOI:** 10.1039/d6ra01843b

**Published:** 2026-07-02

**Authors:** Marina San Miguel Gutiérrez, Francisco Borja Aguirre Yagüe, Ignacio Hernández

**Affiliations:** a Advanced Materials Area, Fundación Centro Tecnológico de Componentes (CTC), Scientific and Technological Park of Cantabria (PCTCAN) 39011 Santander Cantabria Spain; b Department of CITIMAC, University of Cantabria Avenida de Los Castros S/N 39005 Santander Cantabria Spain ignacio.hernandez@unican.es

## Abstract

In this work, the effect of solvent in the luminescence behaviour of nitrogen-doped graphene quantum dots (NGQDs) during their synthesis was evaluated. NGQDs were prepared *via* solvo/hydrothermal methods using citric acid and melamine, as precursors, in a molar ratio of 4 : 3. This study demonstrates that the solvent plays a significant role in tuning the luminescence emission of the synthesised nanomaterials. This modulation is linked to variations in surface functionalization, particularly with regards to the distribution and nature of oxygen- and amine-groups. This behaviour has been confirmed by X-ray photoelectron spectroscopy, and Fourier-transform infrared spectroscopy. Atomic force microscopy analysis showed that the synthesised NGQDs exhibited a graphitic carbon core with an average size centred in the range 2–10 nm, regardless of the solvent used (water, ethanol, acetone, or DMF). Also, the synthesised NGQDs in these solvents exhibited bright visible emission between 450 and 550 nm upon excitation at 365 and 405 nm, respectively. Their performance as selective fluorescence turn-off probes for ferric ions in acidic aqueous medium was evaluated for all synthesis conditions in order to identify the most suitable route to obtain sensitive and efficient luminescent sensors for metal ion detection in acidic conditions.

## Abstract 2

The luminescence behaviour of nitrogen-doped graphene quantum dots (NGQDs) and their iron(iii) detection capabilities in water were studied in view of the solvent employed for their corresponding syntheses.

## Graphical abstract

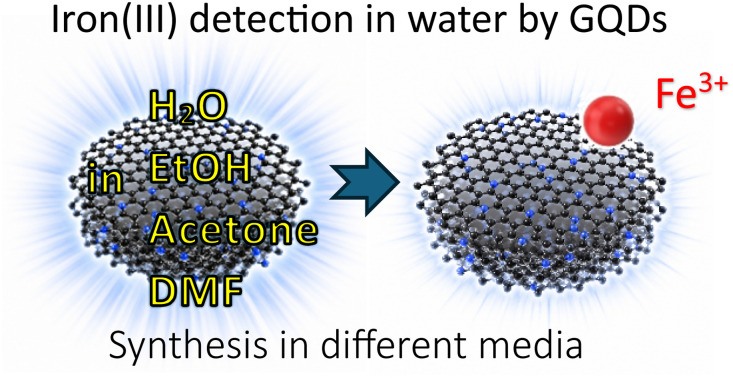

## 1. Introduction

Graphene quantum dots (GQDs) are a class of ultrasmall, photoluminescent carbon nanostructures typically defined by diameters below 10 nm and graphitic sp^2^-carbon cores. Due to their structure, impurity level, quantum confinement and edge effects, GQDs exhibit unique optical and electronic properties, such as size-dependent photoluminescence (PL), exciton confinement or giant red-shift upon varying the excitation conditions.^1^

Over the past decade, extensive research has been conducted to understand and control the photoluminescent behaviour of GQDs. GQDs' PL arises from a combination of factors, including electron–hole recombination, quantum size effects, heteroatom doping, and surface defects. Thence, the optical properties are highly sensitive to the microstructure and morphology of the nanoparticles, which in turn are primarily determined by the synthesis method employed. Various synthetic methods have been explored to tailor the size, shape, and chemical composition of GQDs, including solvothermal, hydrothermal, electrochemical methods, ultrasonic treatment, and pyrolysis of organic precursors.^2,3^

Two broad synthetic methodologies are commonly employed: the “top-down” approach, which involves breaking down bulk carbon materials into nanoscale fragments *via* physical or chemical methods,^4,5^ and the “bottom-up” approach, which assembles GQDs from molecular precursors such as benzene derivatives through processes including pyrolysis, hydrothermal treatment, and stepwise solution chemistry.^6,7^ The bottom-up strategy offers greater flexibility in controlling the elemental composition and physical properties of the resulting nanostructures.

Doping and surface functionalization represent additional tools for tuning the optical and electronic characteristics of GQDs. Among various doping elements, nitrogen has emerged as a particularly effective dopant due to its ability to modulate the electronic density and generate additional active emitting sites. Multiple nitrogen functionalities, such as pyridinic-N, pyrrolic-N, graphitic-N, and amino groups, can be incorporated into the GQDs structure, each of these functionalities having a distinct effect on their physicochemical and optoelectronic properties.^8^ The nature of nitrogen incorporation has a direct impact on optoelectronic behaviour, especially the PL response. In nitrogen-doped graphene quantum dots (NGQDs), the introduction of nitrogen functionalities enhances surface reactivity, improves photostability, and can lead to a redshift in emission due to *n*–π* transitions between nitrogen-containing aromatic structures and the extended π-conjugated domains.^9^

Additionally, changes in quantum confinement and localized π-domains (sp^2^ carbon regions) can modulate the emission wavelength from green to red or even into the near-infrared region. The surface oxygen- and nitrogen-containing functional groups also play a critical role in sensor applications, particularly in metal ion detection.^10^ These functional groups facilitate the energy transfer from the excited NGQDs to the metal ions, leading to fluorescence quenching through the formation of non-radiative ground-state complexes.^11,12^

Recent studies have shown that, sometimes irrespectively of the precursors, the solvents in which the synthesis takes place significantly influence the nature of the NGQDs, particularly in solvo/hydrothermal processes.^13–15^ Solvents can affect the dehydration and carbonization dynamics of organic precursors, modulate the growth of sp^2^-conjugated domains, and influence the incorporation of dopants such as nitrogen into graphitic positions, factors that directly impact the photoluminescence quantum yield.^2,16^ Other studies as Zhang *et al.*^17^ made a comparison between different solvents depending on their boiling point (DMF, water or ethanol). This study demonstrates that solvents with a lower boiling point improved the dehydration and carbonization carbon dots structures promoting their longer wavelength emission.

Aprotic solvents, for instance, have been found to promote a higher degree of carbonization compared to protic solvents. This results in larger π-conjugated domains and redshifted absorption and emission bands.^18^ In this sense, Jianliang Bai *et al.* developed solvent-controlled and solvent-dependent synthesis strategies to produce multicoloured PL carbon dots. This was achieved by adjusting the degree of graphitisation and modifying surface functional groups, using *p*-phenylenediamine and oxalic acid as synthetic precursors.^19^ Using this approach, they successfully synthesised blue and red fluorescent carbon dots under 365 nm UV light excitation, employing ethanol and water as solvents, respectively. The results suggest that the difference in fluorescence colour may be attributed to variations in chemical composition and structure. Similarly, Moon *et al.*^20^ reported that the PL spectra of nitrogen doped carbon nanoparticles synthesised in organic solvents depend on the nature of the solvent employed.

Although there are several studies that provide information about the iron sensing application of GQDs most of them were obtained by hydrothermal technique.^21–23^ Some other studies reported high selectivity GQDs prepared in toluene^24^ for ferric ions detection. This work addresses both the analytical and synthetic dimensions and focuses on the underlying effect of synthesis solvent on sensitivity and selectivity of NGQDs with metallic analytes such as ferric ions. Moreover, it explores the mechanisms leading to the photoluminescence quenching, and their concentration dependence.

The selection reaction media was chosen in terms of different protic and aprotic solvents with different polarities and their potential influence on the fluorescence properties of nitrogen-doped graphene quantum dots during their synthesis. To this end, we investigated how solvent selection affects the luminescence emission and specifically to the Fe^3+^ ions detection of NGQDs synthesised using solvothermal and hydrothermal approaches.

Our findings provide valuable insight for the rational design of highly fluorescent NGQDs through solvent-controlled synthesis, enabling the optimization of their features and enhancing their performance in Fe^3+^ sensing applications. Furthermore, this study investigated the potential of these solvent-controlled NGQDs as fluorescent probes for detecting ferric ions in acidic aqueous solutions and the mechanisms involved. Thus, it was taken into account solvatochromic effects due to polarity, hydrogen bonding and pH of the aqueous medium employed for the detection.

A well-defined, proportional decrease in fluorescence intensity was observed as the concentration of iron ions increased, confirming the potential of these nanomaterials as selective turn-off sensors for these ions in water. In this sense, the sensitivity of these NGQDs enables detection at µM levels, making this system a promising candidate for early-stage corrosion monitoring in acidic environment. This opens exciting possibilities for its application in corrosion detection and remediation across a range of materials.

## 2. Materials and methods

### 2.1. Reagents

Citric acid monohydrate (CA), (99,5%), micronized melamine (99%), *N*′,*N*′-dimethylformamide (DMF), iron(iii) chloride (99%), zinc(ii) chloride (99%), copper(ii) chloride (99%), manganese chloride hexahydrate (99%), nickel chloride hexaydrate (99%) and hydrochloric acid (37%) were purchased from Sigma-Aldrich. Iron chloride anhydrous (99.5%) was purchased from Alfa Aesar and cobalt(ii) chloride hexahydrate from Riedel-de Haën. Ethanol absolute and acetone were purchased from Panreac. Deionized water (0.7 µs cm^−1^) was used throughout the work. All chemicals were used throughout the experiments without further purification processes.

### 2.2. Instruments

X-ray photoelectron spectroscopy (XPS) data were carried out by X-ray photoelectron spectroscopy service in SCAI-UMA facilities using a PHI VersaProbe II equipment. An X-ray source of aluminum (1486.6 eV mono) was employed working at 46.7 W with a 200 µm beam diameter. The Fourier transform infrared (FT-IR) spectra were obtained on an ATR-FTIR Cary 630 spectrophotometer (MicroLab, Agilent). The size distribution of synthesised NGQDs was analysed by dynamic light scattering (DLS) measurement performed by Stabino Zeta (Microtrac, Japan) and Park Systems XE 100 atomic force microscope (AFM) was used to analyse the particle size of the synthesised NGQDs. We employed a Bruker D8 diffractometer with a Cu X-ray tube and a Lynxeye detector for X-ray diffraction experiments.

An Edinburgh FLSP920 spectrofluorometer allowed for the recording of the Emission-Excitation-Spectra (EES) maps and absolute quantum yield at PL maximum for 365 nm excitation (20 nm spectral width). Fluorescence spectral measurements in detection experiments were obtained by a Xenon lamp from a Fluoromax 2 (Horiba) and a Spectral Product SP-245 spectrometer). The excitation wavelength was set to that of widespread UV LEDs, *viz.* 365 nm and 405 nm. 10 Repetitions were taken, with an exposure time of 5 s, the emission wavelength was recorded from 300 to 700 nm with the spectrometer CCD (less than 1 minute total measuring time). The fluorescence intensity at 365 nm was chosen to determine the performance of iron ions detection.

Time resolved fluorescence measurements were obtained using a home-made setup build with a pulsed OPO laser excitation (Q-TUNE-E33, QLI), and a Chromex 500IS spectrometer equipment with a Hamamatsu R928 photomultiplier detector and a Stanford Research SR430 Multichannel scaler.

### 2.3. Synthesis of fluorescence emissive NGQDs

**Fig. 1:**
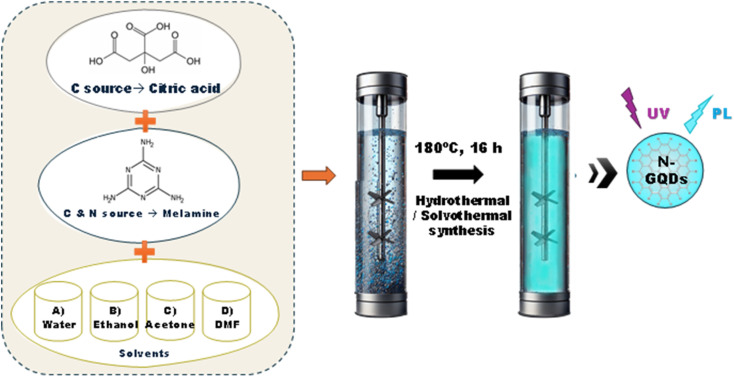
Schematic representation of NGQDs synthesis.

As previously discussed, the NGQDs were synthesised through hydrothermal and solvothermal methods. For that, CA and melamine were dissolved under vigorous stirring with a molar ratio of 4 : 3 in different solvents (water, ethanol, DMF and acetone), see Table S1. The solvents were selected in order to compare the performance of protic and aprotic media of varying polarity during the synthesis process. The precursors and selected solvents were then heated for 16 h at 180 °C in a hydrothermal reactor (Reactor Parr Instruments Mini 4560). Different parameters such as the heating rate, speed of stirring, and temperature were adjusted by PID controller of the hydrothermal reactor. After cooling down naturally to room temperature, the final NGQDs was collected and named as NGQDs_X (X = acetone, DMF, ethanol and water), see Fig. 1. Finally, the NGQDs obtained were filtered using a 0.22 µm microporous membrane. Product yields were around 65%.

### 2.4. Characterization

The characterization of the NGQDs was carried out by the following techniques. Microstructural morphology and size distribution of the NGQDs were evaluated *via* AFM and DLS, as mentioned previously. Also, XPS and FTIR analysis were employed for assessing the chemical composition of the synthesised NGQDs. Thus, XPS measurements were carried out to quantify the presence of different organic moieties were oxygen and nitrogen are involved and to correlate their effect on fluorescence properties of the nanostructures. XRD at Cu K-alpha line was employed to provide evidence of carbonous content.

### 2.5. Iron ion detection

To evaluate the sensitivity of NGQDs as fluorescent probes for iron ions detection, a 2 mL aliquot of NGQDs was incubated with different concentration of iron ions for 2 minutes. The concentration of ferric ions (FeCl_3_) employed ranged from 25 µM to 200 µM acidified with HCl until reach a pH 2 in order to guarantee stability of ferric ion species in solution. Moreover, this work aims at studying the optical properties of GQDs as a sensor in localized pH conditions due to water hydrolysis in pitting corrosion processes in saline media.^25^ The fluorescence intensity was recorded at an excitation wavelength of 365 and 405 nm respectively with the different concentrations of ferric ions. Selectivity assays (excitation wavelength of 365 nm) were carried out with different metal ions (Zn^2+^, Ni^2+^, Co^2+^, Cu^2+^, Ca^2+^, Mg^2+^, Mn^2+^ and Fe^2+^).

## 3. Results and discussion

### 3.1 Morphological and structural characterization of NGQDs

The shape and size distribution of the synthesised NGQDs were analysed by DLS (Fig. S1) and AFM (Fig. S2 and S3) analysis. These techniques also provided an overview of the size distribution of the nanostructures, revealing sizes ranging from 3 to 10 nm. Results using both techniques showed a narrowed size distribution for NGQDs synthesised in the different solvents. No significant differences in size were observed among NGQDs synthesised in aprotic (DMF and acetone) or protic (water) solvents. However, in the case of NGQDs synthesised in presence of ethanol an increase of average size above 10 nm that could be associated to the partial aggregation of particles resuspended in aqueous solution were observed by DLS and topological imaging with AFM. The obtained results are in line with information reported in literature.^2,3,17^

All four samples' X-ray diffractogram (Fig. S4) show a significant content of graphitic/graphenitic carbon in form of a peak at 2*θ* ∼26.5° corresponding to that of (002) interplanar distance, ∼0.34 Å. Besides, those made in organic solvent also show a very broad feature at lower angles, suggesting a portion of amorphous graphene-oxide.^5^ Minor, more crystalline peaks of graphene oxide and maybe others due to unreacted or partly reacted precursors are observed in the samples prepared in water and DMF.

### 3.2 Surface functional group analysis of NGQDs

**Fig. 2:**
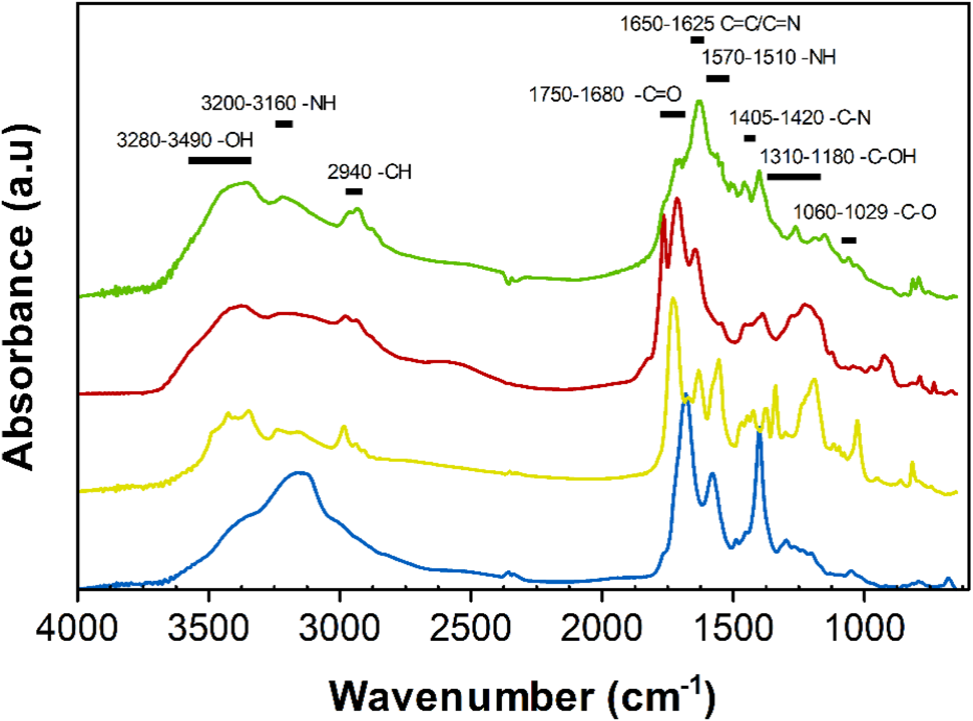
Normalized FTIR spectra of NGQDs synthesised: NGQDs_water (blue), NGQDs_ethanol (yellow), NGQDs_acetone (red) and NGQDs_DMF (green).

The FTIR spectra of synthesized NGQDs are shown in Fig. 2. NGQDs_water, according to its FTIR spectrum, occupies a larger number of polar functional groups such as O–H (3400 cm^−1^), N–H (3200 cm^−1^), C=O (1700 cm^−1^), and C–O (1050 cm^−1^) associated to epoxy groups. Additionally, a significant increase in the C=C/C=N contribution (1625–1650 cm^−1^) was observed in the samples prepared with NGQDs_DMF. This suggests that C=C/C=N and C–N (1405 cm^−1^) contributions are dominant using DMF as solvent media for the synthesis due to the promotion of large conjugated sp^2^ domains^26^ and the content of nitrogen doped heterostructures.^27^ Also, the use of protic (water and ethanol) or even aprotic solvent with a lower dielectric constant (acetone) causes the disappearance of C=C/C=N and a relative increase of other contributions as C=O vibrations of carboxylic (1750 cm^−1^) and amidic (1680 cm^−1^) functionalities.^28^

The signals at 1400 cm^−1^ associated with C–N, 3200 cm^−1^ and 1570 cm^−1^ related to N–H vibrations, inform about the formation of nitrogenated groups on the surface of the graphene structure when solvents with higher polarity are employed (water and DMF). These functional groups are known to promote radiative recombination and can also coordinate with Fe^3+^ ions present in the medium. However, the stretching vibration of N–H decreases as the polarity of solvent employed (NGQDs_water > DMF > ethanol > acetone). In fact, FT-IR spectra obtained showed the weakest contributions of N–H at 3200 cm^−1^ and 1570 cm^−1^ using acetone as reaction medium.

Furthermore, differences were observed in oxidized moieties measured by FTIR. Samples prepared in acetone showed a significant carboxylic contribution (1750 cm^−1^) that decreases in the case of samples synthesized using DMF or water and disappears completely using ethanol as reaction medium. The tendency observed is that solvents with lower polarity reduces the dehydration and carbonization pathways, but aprotic acetone enhance the formation of oxidized carboxylic groups compared to protic ethanol that promotes condensation and insertion of amides. This effect is confirmed by the increase of –N–H contribution (1520 cm^−1^) and C=O signal (1680 cm^−1^) associated with amides and observed in samples prepared with protic solvents. Differences in C–O vibrational modes of epoxy at 1050 cm^−1^ were also detected between protic and aprotic solvents.

XPS analysis provided elemental and chemical state information to complement the FTIR results of the synthesised NGQDs. As can be observed in Fig. S5 XPS spectra carried out indicate the presence of three main signals associated to C 1s, N 1s and O 1s.

The elemental analysis of all samples is represented in Table 1. Measurements of N 1s showed the highest content of nitrogen in NGQDs prepared in the presence of ethanol and DMF. On the other hand, the NGQDs synthesised in presence of acetone showed the lowest content of nitrogen (6.48%). Nevertheless, the total amount of oxygen is similar in every sample except the samples prepared in DMF due to its less oxidative character^20^ and the promotion of dehydration and carbonization. In the case of samples prepared in water the showed a slight reduction of oxygen moieties compared to samples prepared in ethanol an acetone.

**Table 1:** Elemental composition of NGQDs

Samples	C 1s	N 1s	O 1s
N-GQD_water	64.78%	12.70%	22.52%
N-GQD_ethanol	56.89%	18.46%	24.65%
N-GQD_DMF	68.58%	16.26%	15.16%
N-GQD_acetone	58.46%	6.48%	25.90%

**Fig. 3:**
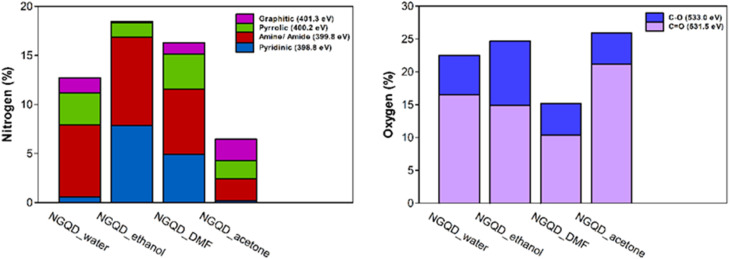
Percentage of nitrogen (left) and oxygen (right) species obtained from deconvolution results of high resolution N 1s and O 1s XPS spectra of synthesised NGQDs.

The high resolution XPS spectra of N 1s (Fig. 3) were deconvoluted into four different signals associated with different nitrogen sites in NGQDs including amine moieties (399.8 eV), pyridinic moieties (398.8 eV) and quaternary also known as graphitic nitrogen (401.3 eV). Pyrrolic moieties (400.2 eV) are overlapped to amine ones, so it is not possible to obtain a reliable quantification of both functionalities. Finally, high-resolution O 1s XPS spectra of NGQDs showed two main deconvoluted signals at 531.5 eV and 533.0 eV attached to C=O and C–O contributions respectively.

According to N 1s XPS results, significant changes were observed in the amount of pyridinic functionality. A reduction in polarity of protic solvent ethanol increases significantly the content of nitrogen in pyridinic positions compared to samples obtained in water. On the other hand, synthesis aprotic acetone showed lowest pyridinic content, but more polar DMF solvent presents an intermediate content of pyridinic groups observed in samples synthesized in water and ethanol. The total amount of pyrrolic and other lateral functional groups (amine/amide) a significant change were measured between DMF and other solvents.

Deconvoluted O 1s curves showed that the use of ethanol and acetone also increases the presence of carbonyl groups but in the case of DMF the contributions of C=O and C–O associated to the presence of epoxy, amide and carboxylic moieties are decreased significantly. This effect confirms the enhancement of dehydration and carbonization pathways observed by FTIR spectroscopy. Moreover, comparing two solvents with similar dielectric constants like ethanol and acetone, results suggest that the use of aprotic solvents slightly decreases the presence of C–O bonds. This same effect was observed in FTIR through a reduction in contribution at 1050 cm^−1^.

### 3.3. Luminescent properties of NGQDs

**Fig. 4:**
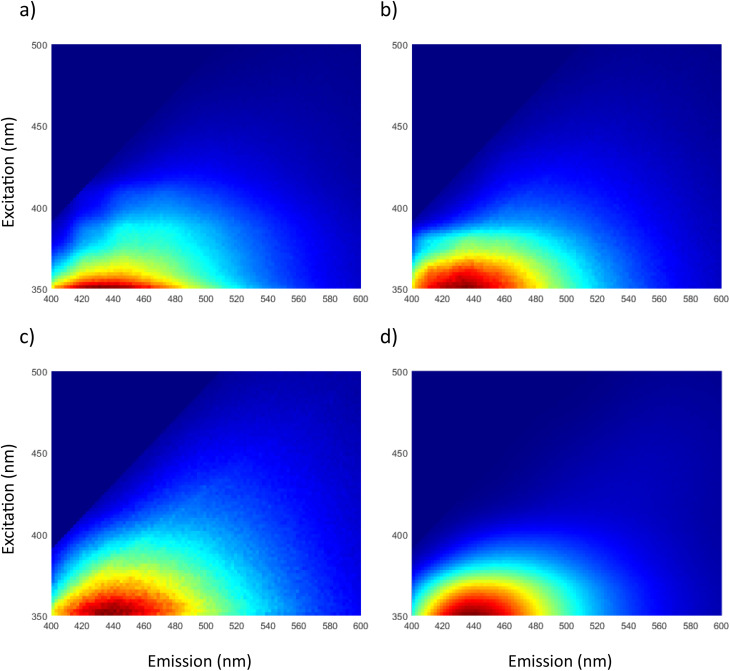
Normalized excitation-emission spectroscopy maps of NGQDs synthesised in different solvents: water (a), ethanol (b), DMF (c) and acetone (d). Max. PL intensity: red, *I* = 0: dark blue. Particles dried and redispersed in water.

The obtained fluorescent samples showed different photoluminescence quantum yields (QY) at excitation and emission maxima, depending on the solvent employed during their synthesis. Samples obtained in aqueous medium (NGQD_water) showed the highest QY at excitation of 43.76%. This value is reduced significantly when reaction medium is substituted by acetone (7.05%), ethanol (4.04%) and DMF (1.43%). Fig. 4 represents the EES maps for the NGQDs samples prepared in different solvents. The typical giant redshift effect upon decreasing excitation energy is clearly observed, the PL shifting to lower energies correspondingly. NGQD_ethanol provides the most redshifted emission and broadest excitation range. All compositions show a somewhat comparable high PL. NGQD_ethanol shows, however, the highest overall intensity, more than five times that of NGQD_acetone and NGQD_water, which in turn are twice as bright as NGQD_DMF. This is due to the contributions of the greatly extended and redshifted tails of both the excitation and emission bands along the samples (Fig. 4). NGQD_ethanol PL intensity is also the highest at commercial 405 nm or 365 nm excitation sources, when computing.

### 3.4. Application of fluorescent GQDs for iron detection

**Fig. 5:**
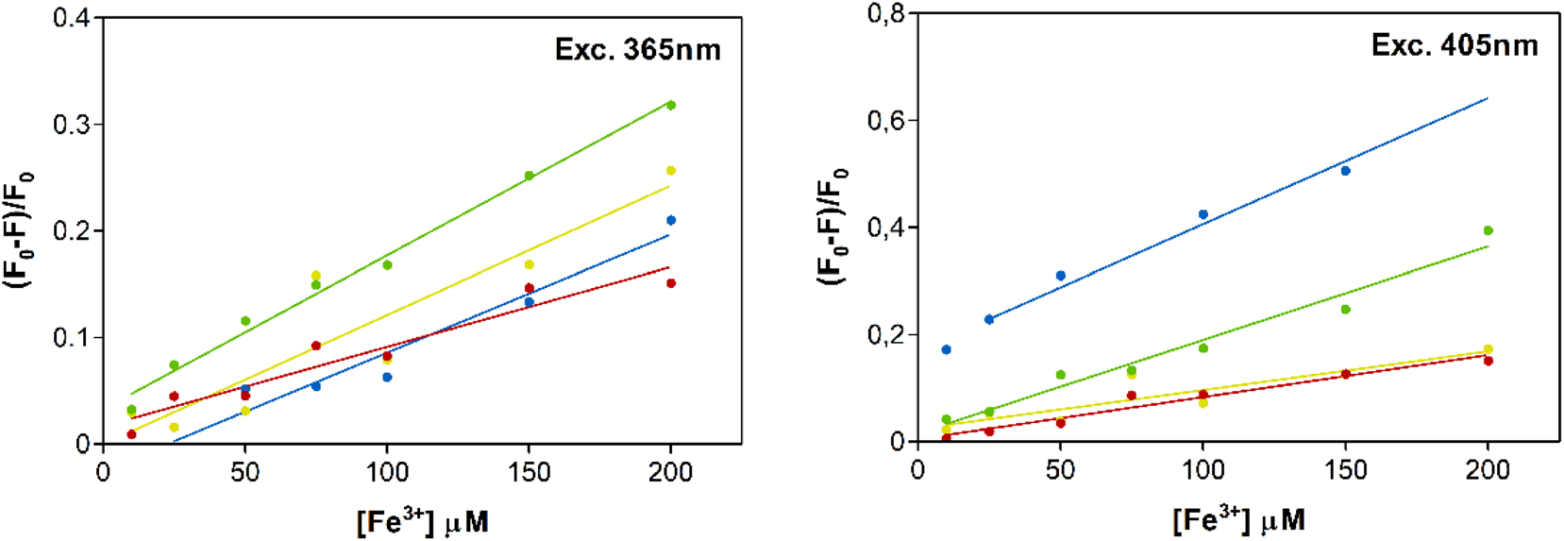
Linear relationship between fluorescence quenching and ferric ion concentration: water (blue), ethanol (yellow), DMF (green) and acetone (red).

To quantify the sensitivity and fluorescence quenching behaviour of the different synthesized NGQDs towards iron ions, their sensing performance was further evaluated over a concentration range of 25–200 µM. The change in normalized fluorescence intensity, *F*, given by (*F*_0_ − *F*)/*F*_0_ gradually increases with concentration of iron ions (as *F* decreases from *F*_0_ at 0 M iron) in all four compositions (Fig. 5).

The limit of detection (LOD) was calculated for iron ions using the equation eqn (1) where *σ* is the standard deviation of the blank, which was determined from 3 blanks.^29,30^ LOD values for each synthesis condition applying two different excitation wavelengths (365 nm and 405 nm) were gathered in Table 2.1
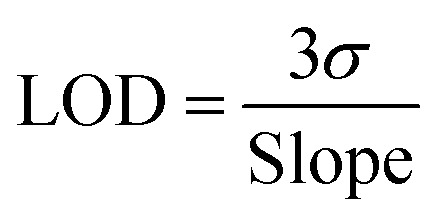


**Table 2:** Analytical performance of synthesised NGQDs

Sample	*λ*_exc_ = 365 nm				*λ*_exc_ = 405 nm			
	Slope (µM^−1^)	*R* ^2^	*N*	LOD_365nm_ (µM)	Slope (µM^−1^)	*R* ^2^	*N*	LOD_405nm_ (µM)
NGQDs_water	0.0011	0.95	3	89	0.0034	0.92	3	66
NGQDs_ethanol	0.0013	0.82	3	20	0.0008	0.84	3	411
NGQDs_DMF	0.0014	0.99	3	29	0.0017	0.97	3	24
NGQDs_acetone	0.0007	0.92	3	552	0.0008	0.96	3	483

LOD results demonstrated a moderate to high sensitivity for iron detection using NGQDs prepared in presence of water and DMF. In the case of ethanol its sensitivity for iron was high at 365 nm excitation wavelength but it decreased significantly exciting with 405 nm light source. The lowest sensitivity was reported by NGQDs prepared in acetone solvent for both excitation wavelengths. Literature provides a wide range of LODs for GQDs in sensing applications. The lowest values we obtain are somewhat higher than those reported for iron detection in bioassays-oriented research,^22^ but reasonable in a context of iron solubility in a corrosive environment.^31,32^

Comparing these results to XPS analysis can be found that the samples prepared in acetone with lowest content of nitrogen presented the highest limit of detection for ferric ions. Besides, samples prepared in water and DMF with a higher content of nitrogen, specifically pyrrolic and pyridinic moieties, showed the most sensitive response. These results confirm previous studies were pyrrolic and pyridinic nitrogen functionalities facilitate the formation of coordination with iron.^33^

Selectivity tests were also carried out employing different 100 µM metal ions (Fig. S8). Results showed in all samples a higher sensitivity to ferric ions compared to other analytes (pH 2). However, NGQD_DMF and NGQD_water showed slight sensitivity to Cu^2+^ and Fe^2+^ respectively. Unspecific interactions with other analytes found for NGQD_DMF could be associated to its structure enriched by pyridinic and pyrrolic moieties and weakened of oxygenated functional groups.

### 3.5. Sensing mechanism

The sensing mechanism of the studied samples in presence of Fe^3+^ ions was elucidated by Stern–Volmer plot analysis (Fig. S9) and PL lifetime decay measurements on the sample before and after the addition Ferric chloride solutions (pH 2) at different concentrations. The PL lifetime was obtained for an excitation at 355 nm, at the emission wavelength of the maximum of the PL spectrum. In these conditions the decay curves are not monoexponential. Two very distinct regimes are observed: a rapid decay curve with a relaxation time in the order of hundreds of nanoseconds and a longer tail lasting for microseconds. The corresponding characteristic lifetimes for these are given in Table 3. Lifetimes do change in the presence of ferric ions. This fact rules out mechanisms such as Primary Inner Filter Effect or intrinsic absorption of nanostructures PL by iron ions, and pin down an actual interaction between the ions and the GQDs. The absence of Inner Filter Effect was confirmed by UV-visible absorption spectra of different samples compared to ferric ion analyte (Fig. S6).

Lifetimes in the absence and in the presence of the analyte, Stern–Volmer constants and energy transfer efficiency of synthesised NGQDs

**Table:** 

Sample	*λ*_ex_ = 405 nm			*κ*_405nm_ (%)
	*τ*_0_ (ns)	*τ*_A_ (ns)	*K*_SV_	
NGQDs_water	446	380	0.0068	14.79
NGQDs_ethanol	310	372	0.0009	—
NGQDs_DMF	300	283	0.0018	5.66
NGQDs_acetone	463	420	0.0009	9.28

**Table:** 

Sample	*λ*_ex_ = 355 nm			
	*τ*_0_^s^ (ns)	*τ*_A50µM_^s^ (ns)	*τ*_A100µM_^s^ (ns)	*τ*_A200µM_^s^ (ns)
NGQDs_water	193	182	174	166
NGQDs_ethanol	215	195	208	207
NGQDs_DMF	214	222	204	330
NGQDs_acetone	193	182	196	180

**Table:** 

Sample	*λ*_ex_ = 355 nm				*λ*_ex_ = 355 nm *K*_SV_
	*τ*_0_^s^ (µs)	*τ*_A50µM_^s^ (µs)	*τ*_A100µM_^s^ (µs)	*τ*_A200µM_^s^ (µs)	
NGQDs_water	10.5	9.5	10.4	10.5	0.0013
NGQDs_ethanol	4.7	4.9	4.9	4.8	0.0016
NGQDs_DMF	5.4	5.1	4.8	6.8	0.0022
NGQDs_acetone	7.0	6.9	6.5	6.2	0.0011

Interestingly, the lifetimes dependence at 355 nm with the analyte concentration is not monotonical for some of the preparations, unlike the PL decrease: for samples NGQD_DMF, for instance, the lifetimes are markedly higher at 200 µM concentration than those at lower concentration. However, a decrease in the average lifetime is observed for these in redshifted excitation (405 nm) and emission (corresponding maxima), except for NGQD_ethanol. Such a behaviour is the fingerprint of a non-pure static or dynamic behaviour,^34^ particularly in the 355 nm excitation regime.

The change in the lifetime obtained with and without the analyte, *τ*_A,_*τ*_0_, respectively for the NGQD_water, NGQD_acetone and NGQD_DMF samples at 405 nm excitation allows deriving the energy transfer efficiency (*κ*), as given by eqn (2). In these conditions, the behaviour is dominated by a single exponential (Fig. S10). The use of eqn (2) involves assuming that all reduction is caused by energy transfer to Fe^3+^-related traps. Thus *κ* account for the interaction between donors present in NGQDs and iron ion based-centres as acceptors during an energy transfer process,^34,35^ which is based on exchange or Förster (FRET) interactions. Protic solvents-synthesised NGQDs marked higher *κ* demonstrating a more intense interaction between the analyte and functional groups in carbon nanostructures.2
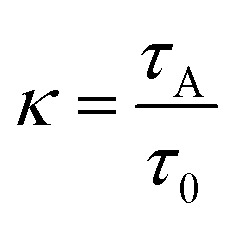


Although the sensitivity analysis involved a relatively narrow range of iron concentrations, the Stern–Volmer plots fitted a linear equation (eqn (3)) over the range 0–200 µM.3
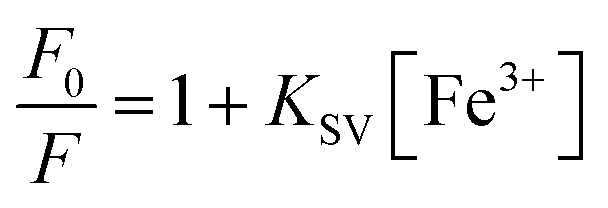


Stern–Volmer constants were gathered in Table 3. Supralinear deviations observed at the higher concentrations confirm a twofold static and dynamic behaviour.

## 4. Conclusion and perspectives

Functional composition and iron sensing capabilities of NGQDs synthesized in different solvent media was investigated to assess the influence of the synthesis solvent on their iron detection properties in water. Samples resulting from all four solvents employed for the particle formation with the same precursors have been characterized. According to FTIR and XPS, DMF and water solvents induce the presence nitrogen-based functionalities such as pyrrolic functionalities.

All four final compositions show giant redshift PL; those made in ethanol providing the most intense and redshifted emission and excitation.

All samples demonstrated significantly and considerably specific interaction with ferric ions resulting in a PL quenching. Selectivity assays showed unspecific interactions with other metallic analytes for samples prepared in DMF. Results obtained by the luminescence assays showed that there are significant differences in the detection of iron analyte between samples prepared in the different solvents that enhance the quenching effect in presence of iron ions. Importantly, the choice of PL excitation energy range, either at 365 or 405 nm, affects sensitivity. DMF and water-mediated synthesis provides the lowest LOD for the 405 nm excitation, while ethanol-mediated ones did at 365 nm.

Lifetime measurements confirmed the Fe^3+^ interaction mechanism that induces the quenching response. However, further analysis should be carried if the dynamic and static components are to be extracted and studied separately. This work is intended as a demonstration for the use of nitrogen doped graphene quantum dots for the detection of iron ions lixiviated during corrosion processes, and the comparison in behaviour upon the synthetic route and presence of different functional groups. It is concluded that these materials are good candidates to be employed as part of PL-probed self-sensing device for corrosion in aggressive media.

## Author contributions

MSM: conceptualization, visualization, methodology, investigation, formal analysis, data curation, writing – original draft preparation. FAY: conceptualization, visualization, methodology, investigation, formal analysis, data curation, writing – original draft preparation. IH: conceptualization, visualization, methodology, investigation, formal analysis, data curation, writing – original draft preparation.

## Conflicts of interest

The authors declare no conflicts of interest.

## Supplementary Material

RA-OLF-D6RA01843B-s001

## Data Availability

The data supporting the reported results in this study are not available upon request from the corresponding author *via* email. Supplementary information (SI) is available. See DOI: https://doi.org/10.1039/d6ra01843b.
