# Supplementary material for: Luminescence behaviour of nitrogen-doped graphene quantum dots via solvent variation for ferric ion sensing
Source: RSC Adv. 2026 Jul 2;16(34):32217–26. doi: 10.1039/d6ra01843b (PMC13325905; doi:10.1039/d6ra01843b)
Supplement: RA-016-D6RA01843B-s001 [file RA-016-D6RA01843B-s001.pdf]

Supplementary Information (SI) for RSC Advances.
This journal is © The Royal Society of Chemistry 2026

# Luminescence behaviour of nitrogen-doped graphene quantum dots via solvent variation for ferric ion sensing

Marina San Miguel Gutiérrez[1], Francisco Borja Aguirre Yagüe[1], Ignacio Hernández[2]

[1]Advanced Materials Area, Fundación Centro Tecnológico de Componentes (CTC), Scientific and Technological Park of Cantabria (PCTCAN), 39011 Santander, Cantabria, Spain

[2] Department of CITIMAC, University of Cantabria, Avenida de los Castros s/n, 39005 Santander, Cantabria, Spain

*Corresponding autor: Ignacio Hernandez; email address: ignacio.hernandez@unican.es.

## Table of content

**Table S1.** Reaction parameters of the NGQDs synthesis.

| Sample | [CA], g | [Melamine], g | Solvent, 450 mL | T, ºC | t, h |
|---|---|---|---|---|---|
| NGQDS_water | 22.69 | 11.34 | Water | 180 | 16 |
| NGQDS_ethanol | 22.69 | 11.34 | Ethanol | 180 | 16 |
| NGQDS_DMF | 22.69 | 11.34 | DMF | 180 | 16 |
| NGQDS_acetone | 22.69 | 11.34 | Acetone | 180 | 16 |

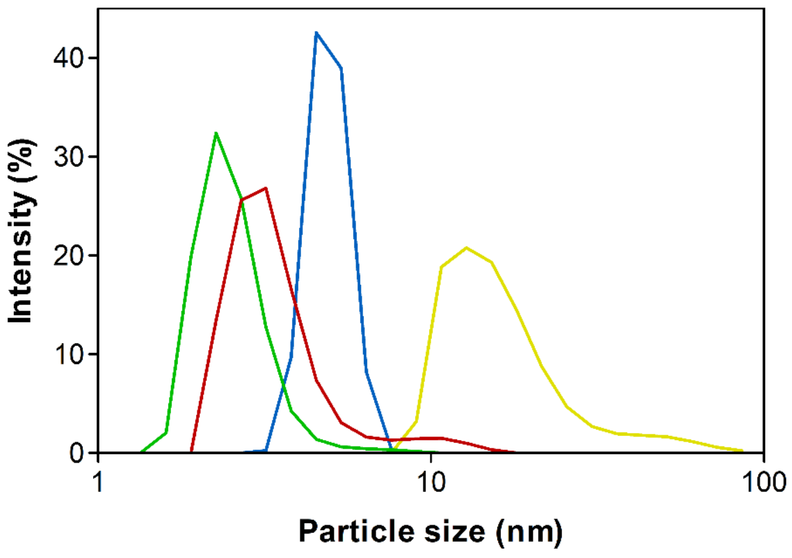


**Figure S1.** Size distribution of synthesized NGQDs determined by DLS analysis: NGQDs_water (blue line), NGQDs_ethanol (yellow line), NGQDs_DMF (green line) and NGQDs_acetone (red line).

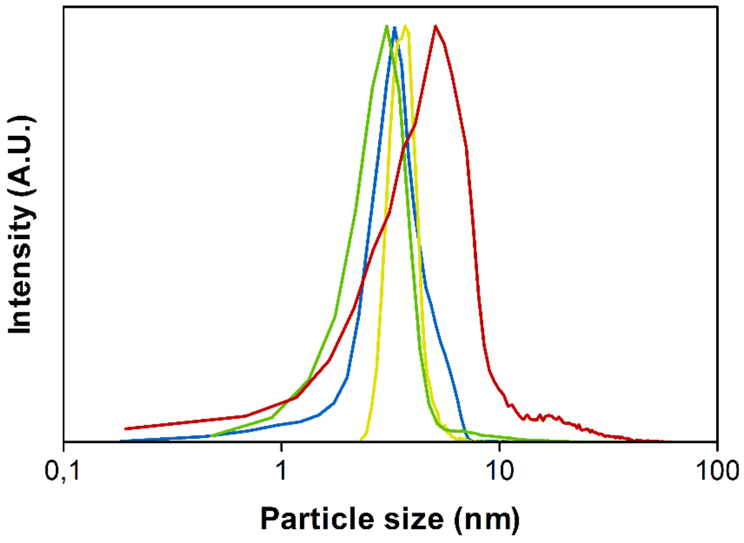


**Figure S2.** Normalized size distribution of synthesized NGQDs determined by AFM analysis: NGQDs_water (blue), NGQDs_ethanol (yellow), NGQDs_DMF (green) and NGQDs_acetone (red) obtained using AFM.

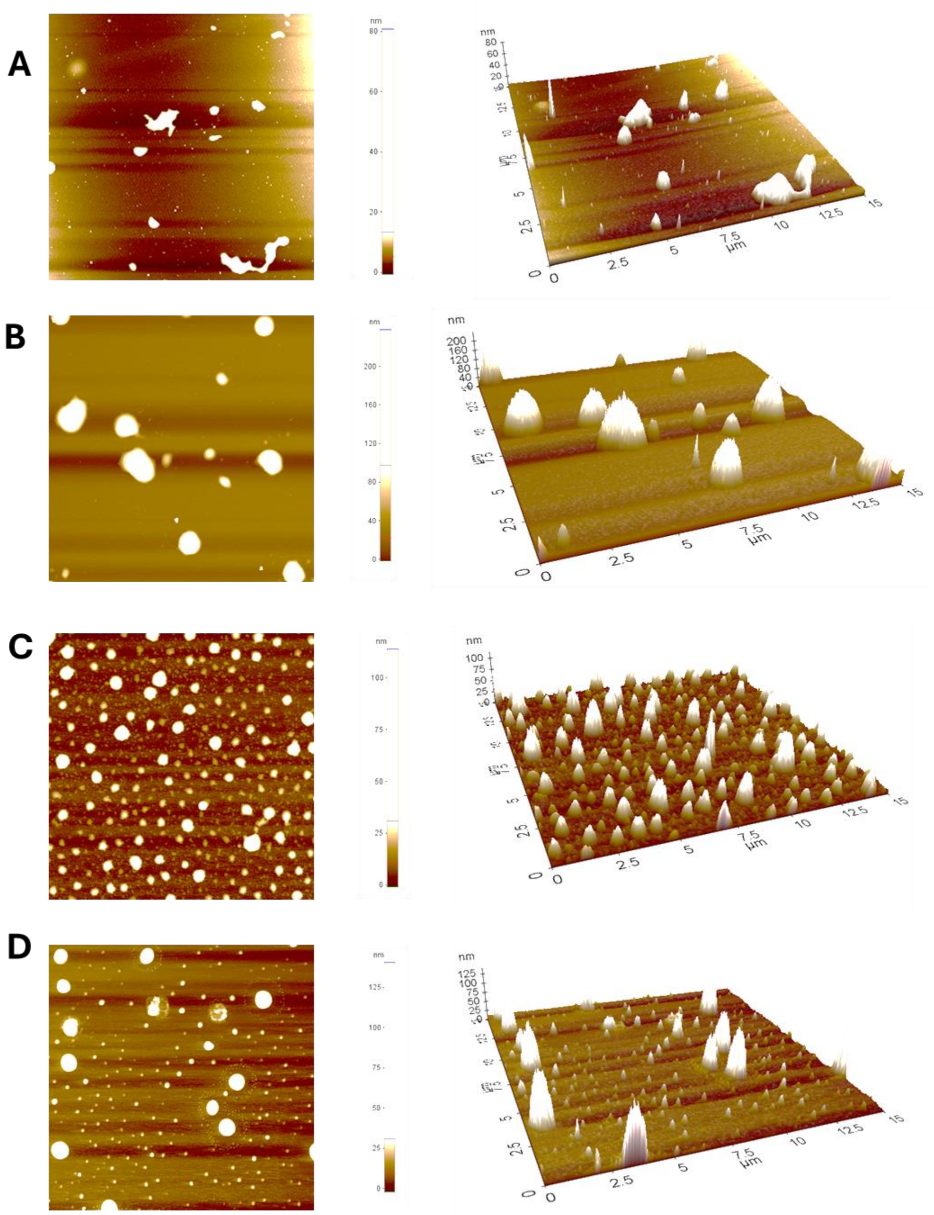


**Figure S3.** AFM topography maps of A) NGQDs_water, B) NGQDs_ethanol, C) NGQDs_DMF and D) NGQDs_acetone.

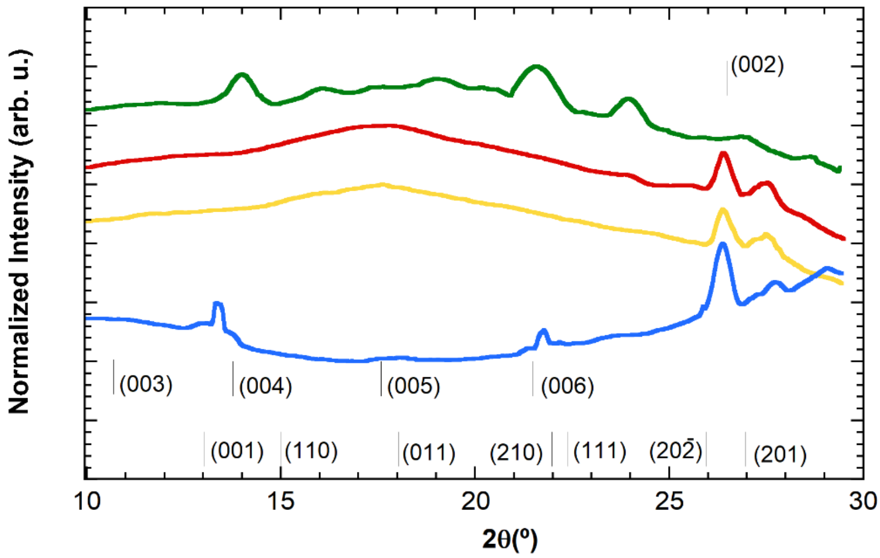


**Figure S4.** X-ray diffractogram for NGQDs_water (blue), NGQDs_ethanol (yellow), NGQDs_DMF (green) and NGQDs_acetone (red) obtained using $\lambda$ = 1.541 Å. The vertical lines represent representative peaks of Graphite/Graphene (on top of the graphs), graphene oxide (top, under the graphs) and 1,3,5-triazine-2,4,6-triamine (melamine, bottom, under the graphs), and numbers are corresponding Miller indexes. COD structure identifiers for graphene oxide and melamine: 1568391 and 2205105, respectively

**Table S2.** Elemental composition of NGQDs.

| XPS Deconvolution | | NGQD_water | NGQD_ethanol | NGQD_DMF | NGQD_acetone |
|---|---|---|---|---|---|
| C 1s | C-C/C-H (284.8 eV) | 63.49% | 35.44% | 57.22% | 60.67% |
| | C-O/C-N/C-O-C (286.1 eV) | 10.77% | 21.64% | 17.07% | 15.76% |
| | R-C=O (287.2 eV) | 2.85% | 10.65% | 12.29% | 4.41% |
| | O-C=O (288.4 eV) | 22.89% | 32.26% | 13.42% | 19.15% |
| O 1s | C=O (531.5 eV) | 73.10% | 60.55% | 68.40% | 81.84% |
| | C-O/water (533.0 eV) | 26.90% | 39.45% | 31.60% | 18.16% |
| N 1s | Pyridinic (398.8 eV) | 4.61% | 42.54% | 30.37% | 3.15% |
| | -NH2/O=C-NH2 (399.8 eV) | 57.67% | 48.85% | 40.55% | 34.08% |
| | Pyrrolic (400.2 eV) | 25.62% | 7.84% | 22.15% | 28.50% |
| | Quaternary/Graphitic (401.3 eV) | 12.10% | 0.76% | 6.93% | 34.27% |

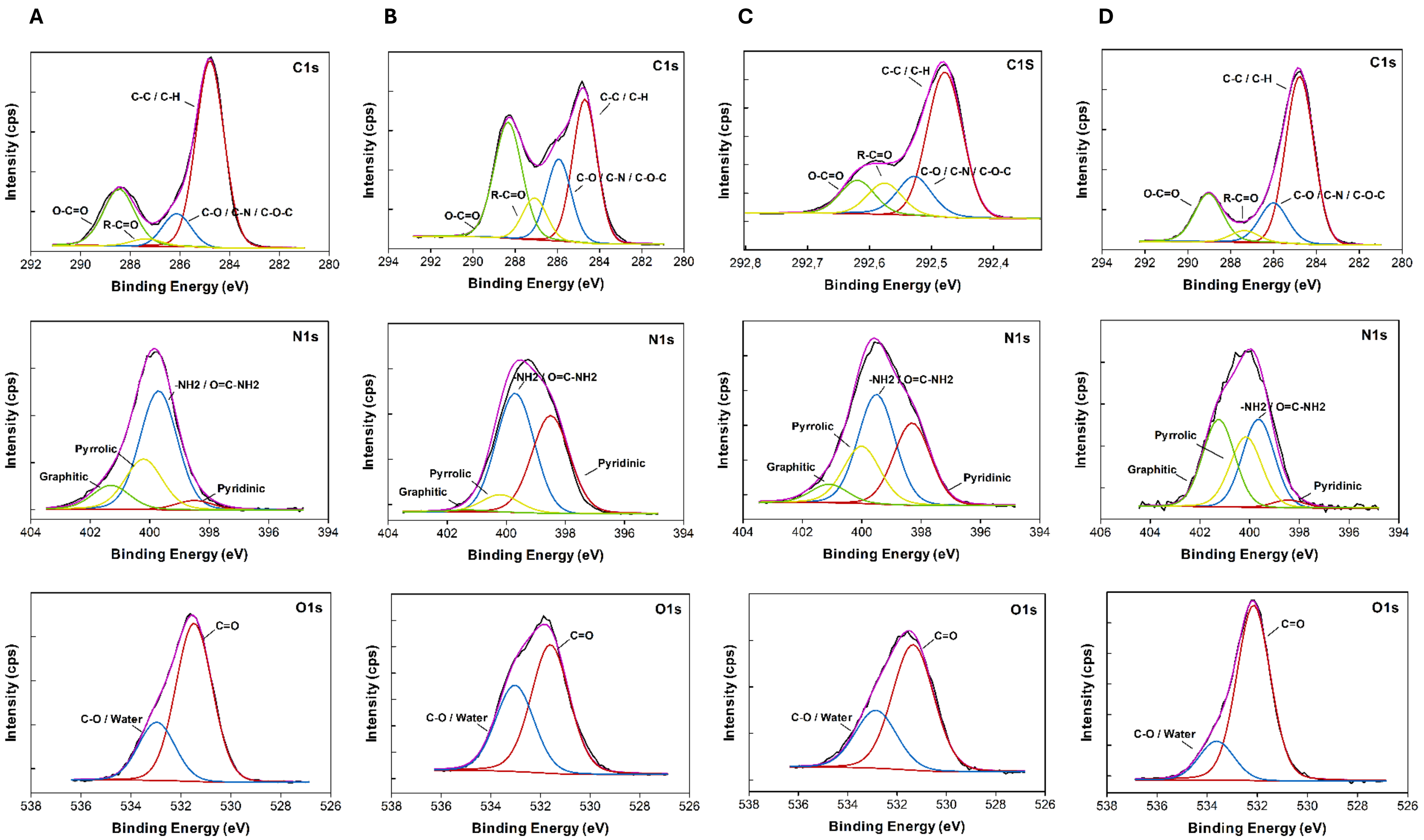


**Figure S5.** High Resolution XPS spectra of synthetized GQDs by solvothermal and hydrothermal treatment. A) NGQD_water, B) NGQD_ethanol, C) NGQD_DMF, and D) NGQD_acetone

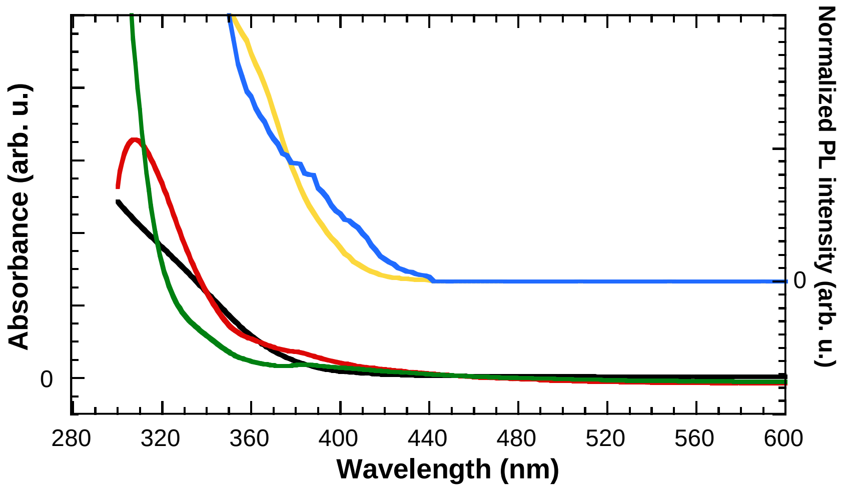


**Figure S6:** UV-Visible absorption spectra of $Fe^{3+}$ (black curve), NGQD_DMF (green) and NGQD_acetone (red), and PL excitation spectrum of NGQD_water (blue), NGQD_ethanol (yellow). Due to the very enhanced emission spectrum NGQD_water, NGQD_ethanol, artifacts hamper a correct determination of the absorption edge.

**A**

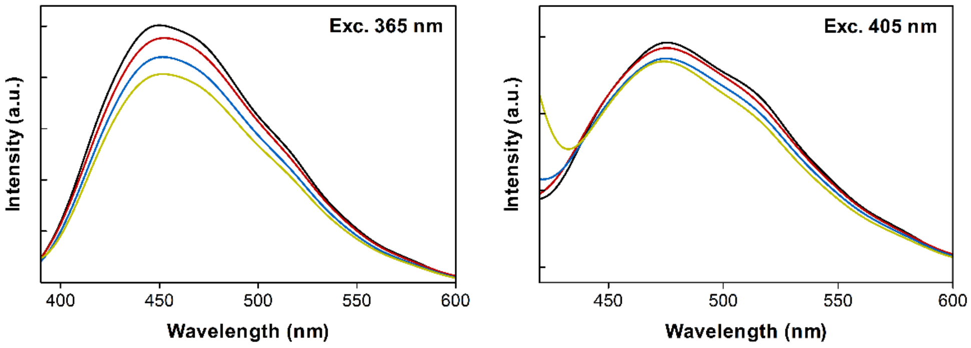


**B**

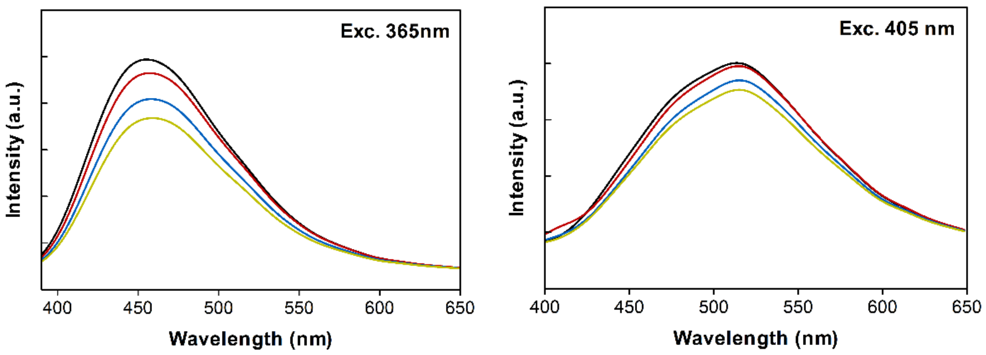

C

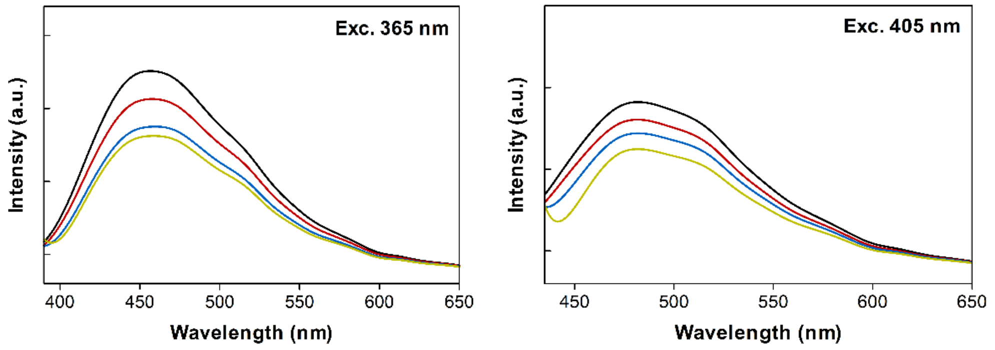


D

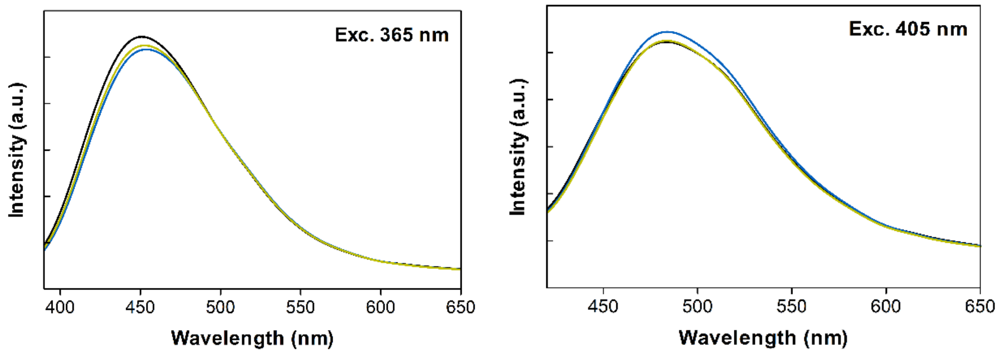


**Figure S7.** PL spectra of NGQDs prepared in A) water, B) ethanol, C) DMF and D) acetone with different concentrations of $Fe^{3+}$: 50 μM (red), 150 μM (blue) and 200 μM (yellow).

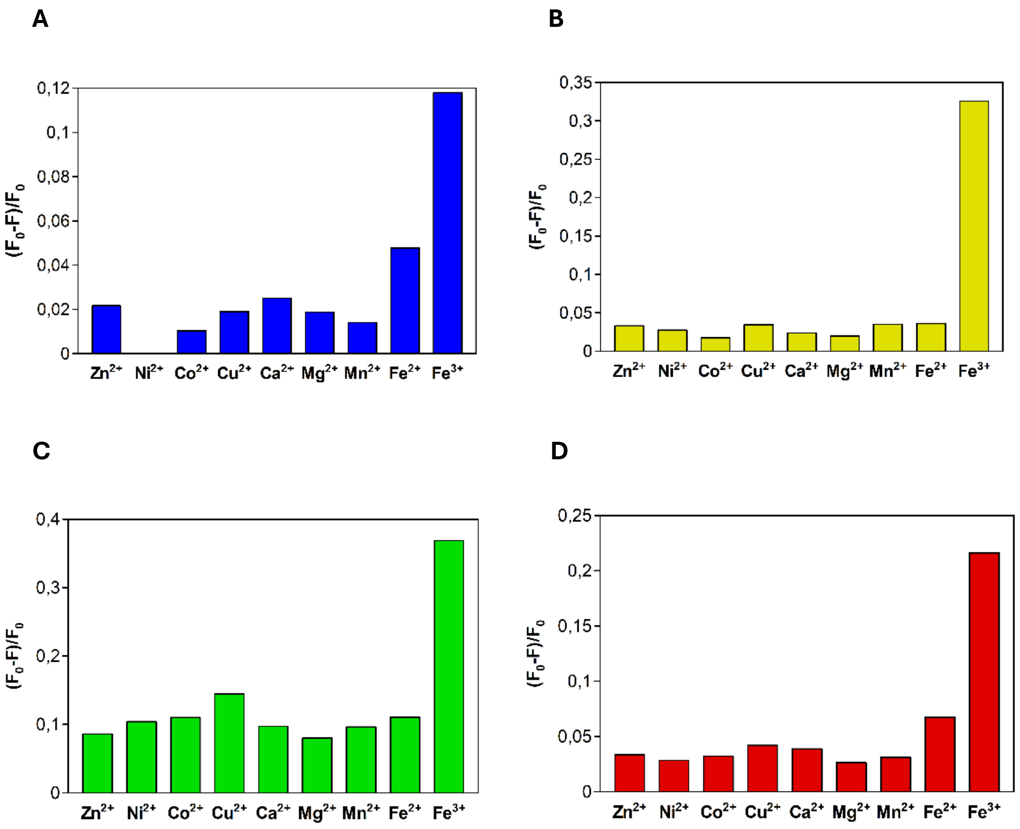


**Figure S8.** Selectivity of NGQDs prepared in A) water, B) ethanol, C) DMF and D) acetone in presence of different analytes at 100 μM (pH 2) and 365nm excitation wavelength.

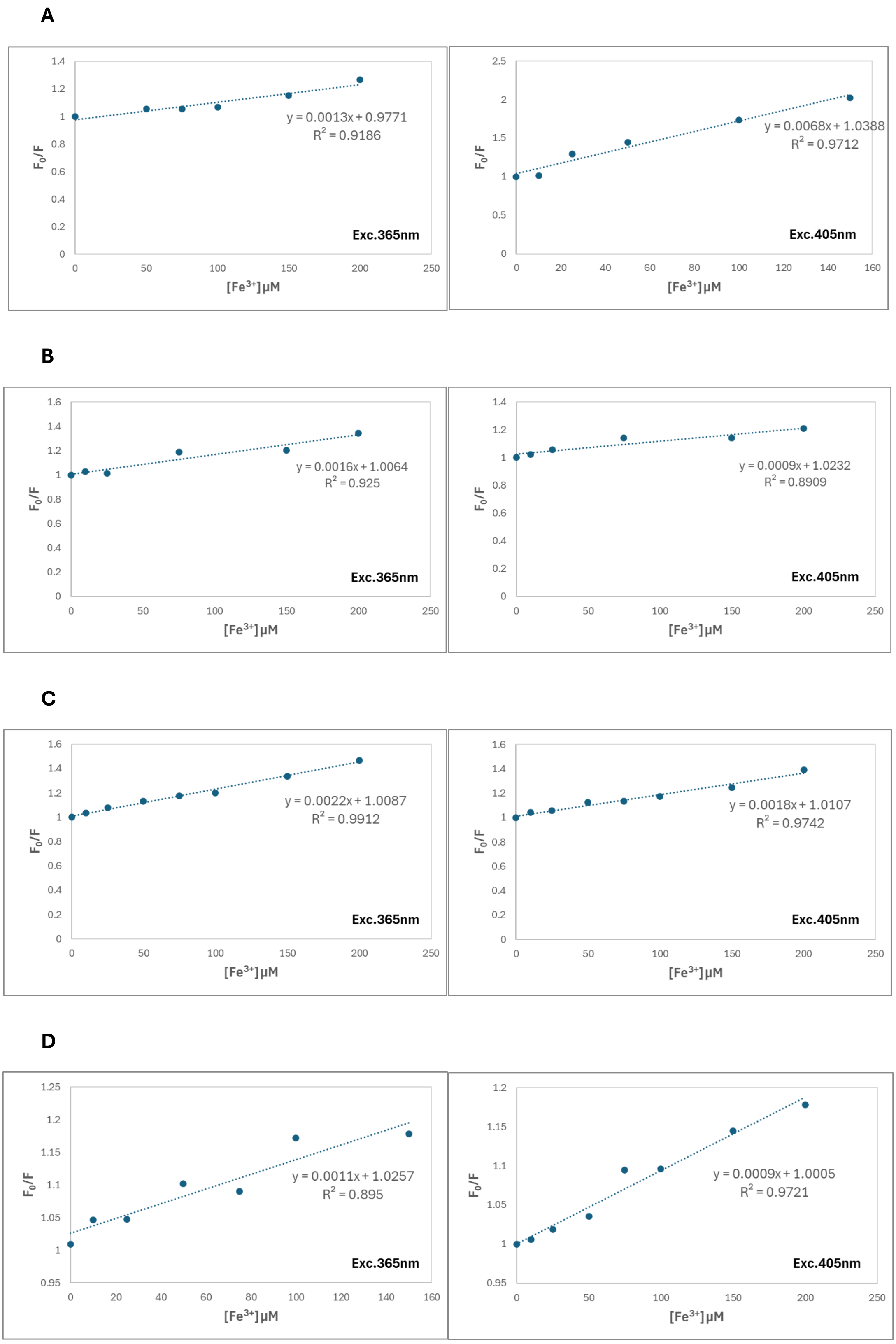


**Figure S9.** Stern Volmer plots and linear regressions of sample synthesized in A) water, B) ethanol, C) DMF and D) acetone suspended in water.

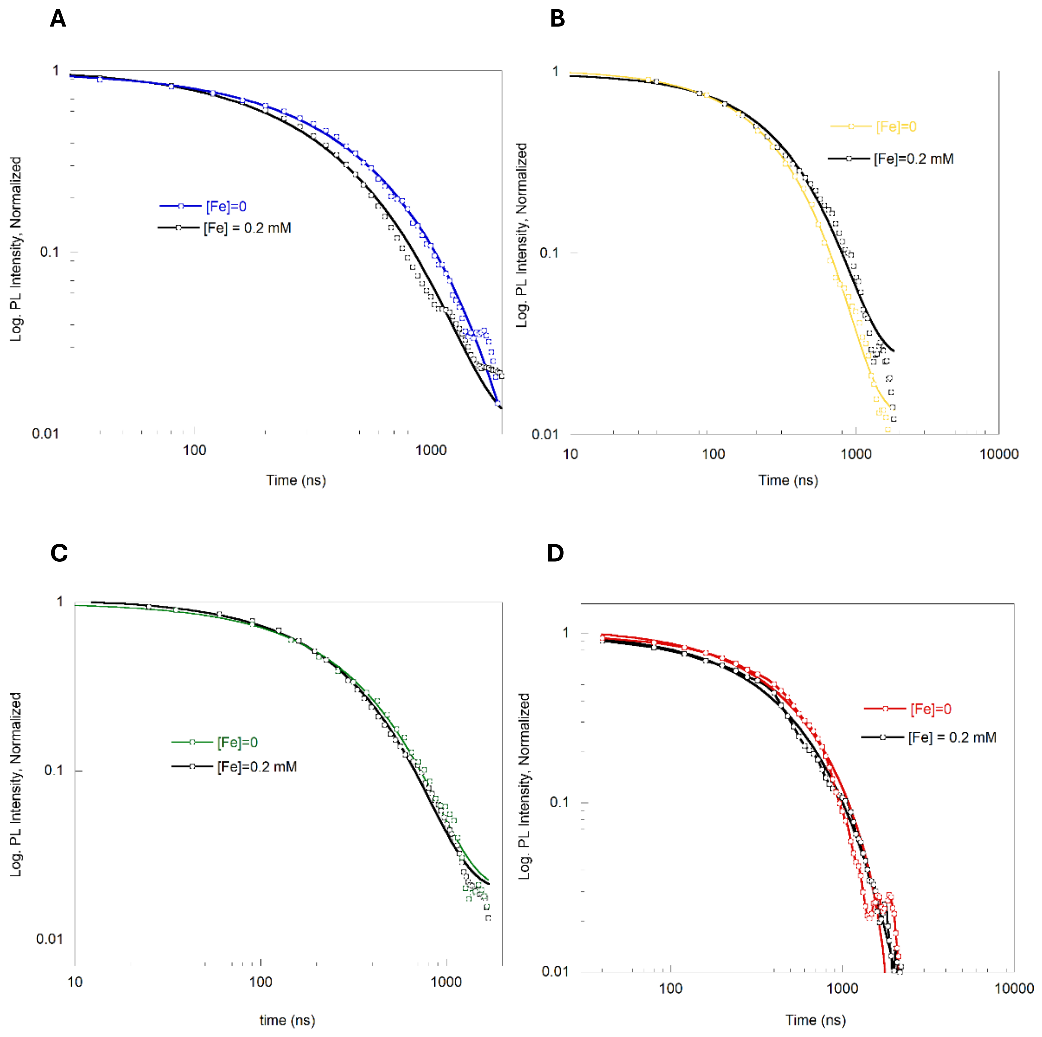


**Figure S10.** Time decay measurements of NGQDs prepared in A) water, B) ethanol, C) DMF and D) before and after addition of $Fe^{3+}$ 200 μM at 405 nm excitation.
